# Effects of packaging and storage conditions on the quality of amoxicillin-clavulanic acid – an analysis of Cambodian samples

**DOI:** 10.1186/2050-6511-14-33

**Published:** 2013-06-18

**Authors:** Mohiuddin Hussain Khan, Kirara Hatanaka, Tey Sovannarith, Nam Nivanna, Lidia Cecilia Cadena Casas, Naoko Yoshida, Hirohito Tsuboi, Tsuyoshi Tanimoto, Kazuko Kimura

**Affiliations:** 1 Drug Management & Policy, Kanazawa University, Kakuma-machi, Kanazawa, Ishikawa 920-1192, Japan; 2 Médecins Sans Frontières, 14 Sayat-Nova street, Vanadzor, Lori, Armenia; 3 Department of Pharmacy, Saiseikai-Nakatu Hospital, Shibata, Osaka 530-0012, Japan; 4 National Health Product Quality Control Center, Ministry of Health, Phnom Penh, Cambodia; 5 Becton Dickinson de México, S.A.de C.V. Km 37.5 – Aut. México-Qro. Parque Industrial Cuamatla, Cuautitlán Izcalli., Edo. De México, CP 54730, México; 6 Department of Analytical Sciences, Faculty of Pharmaceutical Sciences, Doshisha Women’s College, Kyoto, Japan

**Keywords:** Medicine quality, Tropical country, Public health, Packaging condition, Substandard medicine, Developing country

## Abstract

**Background:**

The use of substandard and degraded medicines is a major public health problem in developing countries such as Cambodia. A collaborative study was conducted to evaluate the quality of amoxicillin–clavulanic acid preparations under tropical conditions in a developing country.

**Methods:**

Amoxicillin-clavulanic acid tablets were obtained from outlets in Cambodia. Packaging condition, printed information, and other sources of information were examined. The samples were tested for quantity, content uniformity, and dissolution. Authenticity was verified with manufacturers and regulatory authorities.

**Results:**

A total of 59 samples were collected from 48 medicine outlets. Most (93.2%) of the samples were of foreign origin. Using predetermined acceptance criteria, 12 samples (20.3%) were non-compliant. Eight (13.6%), 10 (16.9%), and 20 (33.9%) samples failed quantity, content uniformity, and dissolution tests, respectively. Samples that violated our observational acceptance criteria were significantly more likely to fail the quality tests (Fisher’s exact test, p < 0.05).

**Conclusions:**

Improper packaging and storage conditions may reduce the quality of amoxicillin–clavulanic acid preparations at community pharmacies. Strict quality control measures are urgently needed to maintain the quality of amoxicillin–clavulanic acid in tropical countries.

## Background

Medicine plays an important role in maintaining health, preventing disease and saving lives. However, ineffective medicines pose great risks to individuals and even threaten lives in emergencies [1,2]. Ineffectiveness takes several forms, such as medicines containing less than the stated dose of the active ingredient or containing unstated or harmful substance(s). Similarly, fake or counterfeit medicines and medicines that have been degraded or adulterated due to improper storage and handling may be ineffective [3,4]. Hence, the health ministries of many countries, especially developing nations, struggle to prevent the circulation of substandard and counterfeit medicines [5-10].

A combination of amoxicillin and clavulanic acid was introduced in the United Kingdom in 1981 as Augmentin and eventually became the treatment of choice for many infections [11,12]. Amoxicillin–clavulanic acid is available in a variety of doses: 250/125 mg (2:1), 500/125 mg (4:1), 875/125 mg (7:1), 1000/125 mg (8:1), and 2000/125 mg (16:1). In combined preparations, 125 mg of clavulanic acid is sufficient to inhibit β-lactamase–producing organisms. Amoxicillin–clavulanic acid also has proven more effective for the eradication of *H. pylori* than conventional monotherapies [13,14]. However, insufficient doses and inappropriate use of such potent antibiotics may lead to the development of resistance [15].

Several studies have reported the presence of substandard and counterfeit medicines in Cambodian pharmaceutical markets, with prevalences ranging from 4% to 90% [5,7]. Several of these studies suggest that antibiotics are deliberately counterfeited in some cases but unintentionally degraded in others [7,16]. The improper storage and handling of medicines in tropical countries may cause the unintentional degradation of medicines [17]. Based on previous studies in Cambodia, the Cambodian Ministry of Health (MoH), and Kanazawa University decided to conduct a collaborative study of the quality of amoxicillin–clavulanic acid in the private pharmaceutical market under tropical conditions in a developing country [7,9].

## Methods

### Selection of medication and study area

Combination tablets of amoxicillin–clavulanic acid were selected from the essential medicine list of Cambodia in consultation with the country’s MoH. Of the various formulations of amoxicillin-clavulanic acid, only tablets appear on the essential medicine list of Cambodia. Because this study did not involve human subjects, ethical clearance was not sought. However, a memorandum of understanding was signed by the Cambodian MoH before commencement. Equal numbers of samples were collected from urban and rural areas. Seven districts of the capital (Phnom Penh) were selected to represent urban areas, and three provinces (Kandal, Takeo, and Kampong Speu) were selected to represent rural areas. The locations were selected after taking into account population density, the number of outlets, and budgetary limitations. The selections were made in consultation with the Department of Drugs and Food and the National Health Product Quality Control Center.

### Collection of samples

Sampling was conducted in July-August 2009 by two teams. Each team consisted of three members: a researcher, a locally recruited supervisor and an assistant. All members of the sampling teams were provided with training beforehand and instructed to pose as typical customers. Stratified random sampling was used to collect samples from four types of private drug outlet (Pharmacy, Depot-A, Depot-B and nonlicensed outlets). A sampling form was completed for each sample after payment. Each sample was then labeled with a code number and stored at 20-25°C until analysis.

### Sample analysis

#### Observational analysis

Primary and secondary packaging and printed labels were carefully observed with the naked eye at the Department of Drug Management and Policy, Kanazawa University, Japan. Samples were classified into five types according to package type and the presence of desiccants (e.g., silica gel):

Type A: Press-through packaging (PTP) of aluminum-aluminum materials in cardboard boxes.

Type B: Type A tablets wrapped in transparent plastic with silica gel.

Type C: Type A tablets wrapped in aluminum with silica gel.

Type D: Similar to Type C, but with PTP made of an aluminum-plastic composite.

Type E: Strip packaging (SP) in cardboard boxes without silica gel.

Samples having any of the following packaging defects were considered unacceptable: 1) PTP/SP packaging with peeling of the cover; 2) missing tablet(s); 3) PTP/SP without any clear pocket breaks.

#### Authenticity

A database of manufacturer addresses was prepared using labels, online searches, e-mail and telephone communication. Portions of all samples were sent to the manufacturer with a request for authentication. Furthermore, the medical regulatory authorities (MRAs) of the manufacturers’ countries were queried on the legitimacy of the manufacturers and their products. Taking into consideration the WHO definition of counterfeit medicines, all information was then cross-checked to arrive at a final determination on the authenticity of the samples and their manufacturers [9,18].

#### Chemical analysis

Dosage and uniformity tests were conducted on 10 tablets according to the United States Pharmacopoeia (USP 30) by high-performance liquid chromatography. A Shim-pack CLC-ODS (M) 15 cm column (Shimadzu, Kyoto, Japan) was used. Dissolution tests were conducted on 6 tablets for each sample using an NTR-VS6P dissolution tester (Toyama, Osaka, Japan) according to USP 30. For quantity tests, tablets were expected to contain 90-120% of the labeled dose. The maximum value accepted in the content uniformity test was 15.0. For dissolution tests, ≥ 85% of the labeled dose of amoxicillin, and ≥ 80% of the labeled dose of clavulanic acid was expected to dissolve in 30 minutes, respectively [19,20].

A stability test was conducted on amoxicillin/clavulanic acid at 37°C with 100% relative humidity (RH). We used one control sample with no visible defect, one with torn wrapping but no strip defect, and one in which the strips were deliberately perforated. In all three samples, amoxicillin and clavulanic acid contents were measured at 0, 24, 48, 72, and 96 hours. These times were based on an exploratory experiment: significant degradation of amoxicillin was observed within 24 hours under tropical conditions (37°C, 100% RH) (unpublished report).

### Statistical analysis

Data analysis was performed using SPSS version 17.0.0 (SPSS Inc., Chicago). When appropriate, Fisher’s exact test and the Bonferroni multiple t-test were used to test the significance of differences in categorical and quantitative variables, respectively. Statistical significance was evaluated at the 5% level.

## Results

### Medicine outlets

A total of 59 samples were collected from 48 outlets in Phnom Penh and surrounding provinces. Of these samples, 26 (44.1%) were collected from Phnom Penh, and the rest from the provinces. Forty-eight (81.4%) of the samples were from licensed outlets (31 (52.5%) Pharmacy, 4 (6.8%) Depot A and 13 (22%) Depot B); the rest (11/18.6%) were from unlicensed outlets. At least one pharmacist was found to be present in 14 of the pharmacies. Air conditioning was present in only one pharmacy. A Cambodian registration number was found on 93.2% of the samples. No sample was past the expiration date.

### Package condition

According to the printed information, most of the samples (55, 93.2%) were imported. Only four (6.8%) samples were manufactured domestically. Fourteen (23.73%) of the samples were branded, and the rest (45, 76.27%) were generic. Fifty-seven (96.6%) of the samples were of 500 mg/125 mg strength, and the rest were 875 mg/125 mg. The labels on 16 samples recommended storage below 25°C; most others simply recommended avoidance of humid conditions. On one product, storage below 15°C was recommended.

On observation of all samples, 32 (54.2%) from six manufacturers were categorized as type A, six (10.2%) from one manufacturer as type B, 2 (3.4%) from one manufacturer as type C, eight (13.6%) from four manufacturers as type D and 11 (18.6%) from four manufacturers as type E (Table 1). We found 12 (20.3%) samples with defective packaging. Two samples had the plastic covers peeled off the strips, one had a strip with missing tablets, and nine had an unclear scored line of package break for individual tablets (Table 1). There was a significant association between the presence of instructions on humidity and the pass rate in the observational test (p < 0.05). No significant association was found between the results of the observational test and any of the following parameters: sampling location, shop category, registered or unregistered store, country of origin, branded/generic product or condition of packaging (intact or sealed and unsealed or open).

**Table 1:** Results of observational tests

**Type**	**No. of manufacturers**	**No. of samples**	No. of samples with packaging defects*			**No. of total failed samples**	**(%)**
			**a**	**b**	**c**		
A	6	32	2	0	4	6	18.8
B	1	6	0	0	3	3	50.0
C	1	2	0	0	0	0	0.0
D	4	8	0	0	0	0	0.0
E	4	11	0	1	2	3	27.3
Total	16	59	2	1	9	12	20.3

* Identified packaging defects:

a = PTP/SP packaging with peeling of the outer plastic or aluminum cover.

b = missing tablet(s) in intact PTP or SP.

c = PTP/SP without clear pocket breaks.

### Authenticity

Requests were sent asking 15 manufacturers to authenticate 57 products. One manufacturer could not be contacted. Six manufacturers responded for 28 (49.1%) of the samples. All of these samples were described as authentic. Only three of 11 MRAs replied to our requests for verification of the legitimacy of manufacturers and samples.

### Quality analysis

Of 59 samples, eight (13.6%), 10 (16.9%), and 20 (33.9%) failed the dose, content uniformity and dissolution tests, respectively. No significant relationship was observed between failure rate and area of collection, shop category, registration status, origin, branded/generic product type, intactness of packaging (i.e., sealed or opened) or response to authenticity investigation. However, failure rates in the quality tests were significantly associated with the outcome of observational analysis (Fisher’s exact test, p < 0.05; Table 2).

**Table 2:** Comparison of quality tests with observation

		**Quantity test (n = 59)**		**Fisher’s exact test**	**Content uniformity (n = 59)**		**Fisher’s exact test**	**Dissolution test (n = 59)**		**Fisher’s exact test**	**Failed ≥ 1 test (n = 59)**		**Fisher’s exact test**
		**Passed**	**Failed**		**Passed**	**Failed**		**Passed**	**Failed**		**Passed**	**Failed**	
Condition of packaging	No defects	46	1	p < 0.05	44	3	p < 0.05	35	12	p < 0.05	33	14	p < 0.05
	Defects	5	7		5	7		4	8		2	10	

**Figure 1:**
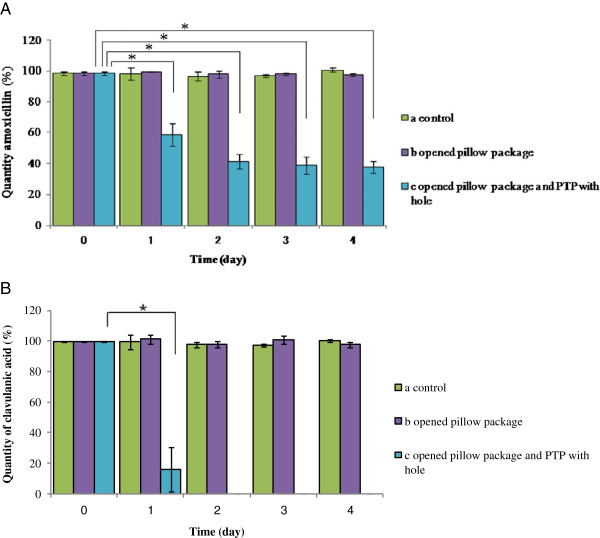
**Changes in concentrations of amoxicillin and clavulanic acid (n = 3) in each packaging condition during storage.** Each value represents the mean ± SD of three experiments. *Significant difference (p < 0.01) compared to baseline using Bonferroni’s multiple t-test. **A**. Changes in the concentration of amoxicillin in tablets (n=3) with each packaging condition during storage (**A**). Each value represents the mean ± SD of three experiments. *Significant difference (p<0.01) compared to baseline using Bonferroni’s multiple t-test. **B**. Changes in the concentration of clavulanic acid in tablets (n=3) with each packaging condition during storage (**B**). Each value represents the mean ± SD of three experiments. *Significant difference (p<0.01) compared to baseline using Bonferroni’s multiple t-test.

Interestingly, clavulanic acid accounted for most failures in the quantity and content uniformity tests (87.5% and 70% of failures, respectively), whereas amoxicillin accounted for most failures (80%) in the dissolution tests. Most of the samples (7 of 8 and 9 of 10, respectively) that failed the quantity and uniformity tests, as well as all (20) samples that failed the dissolution tests, were generic. The results of the stability test suggest that both amoxicillin and clavulanic acid decompose significantly within one day (Bonferroni’s multiple t-test, P < 0.05, Figure 1).

## Discussion

### Medicine outlets

Public medical services in Cambodia still struggle with a lack of medical staff in hospitals, and there are other problems such as long waiting times and high costs [21]. The sale of antibiotics without a prescription for the treatment of mild symptoms is very common at pharmacies and clinics [22]. Seeking health care in the private sector as a first choice is widely observed in many developing countries [23]. Our study found a prescription regulation warning on the packaging of less than half (40.7%) of the samples. The inappropriate use of antibiotics without prescription may promote resistance to even the newest and most effective antibiotics [24]. Sample collection was conducted in July-August, when average daytime temperatures were 30-40°C with high humidity. Although there were recommended storage conditions in the package inserts of several samples, air conditioning was found in only one pharmacy out of 54 outlets. Medicines that are rapidly degraded in adverse environments should not be supplied by outlets that cannot comply with storage requirements [25,26].

### Condition of packaging

Pharmaceutical packaging is an important part of the overall manufacturing and distribution process. Good packaging can ensure the quality and effectiveness of its contents [27]. Several of the samples in this study had no clear-cut break lines between tablets; thus, adjacent tablets may be exposed to air when a tablet is torn from a strip. Two samples that failed the observational test due to peeling of the outer plastic or aluminum cover might have been affected by improper handling during distribution. One sample that failed due to missing tablets in intact PTP and 9 samples that failed due to indistinct pocket breaks may have resulted from poor manufactu ring practices. Ten of these samples also failed one of the quality tests. Hence, these 10 samples are not counterfeit but rather degraded authentic products. Strict adherence to good manufacturing, distribution, and pharmacy practices may improve the quality of medicines for end users [27].

### Authenticity

This study received few responses from MRAs and manufacturers, which may be due to the methodology used in the authenticity investigation. This issue is a low priority for manufacturers and MRAs [7]. Failed samples were attributed to degradation; no counterfeit products were identified. Nevertheless, cooperation from all stakeholders is crucial to safeguard medicine quality, especially from counterfeits [7,28].

### Quality analysis

Samples that failed the content uniformity test usually came from open packages/containers during sampling (Fisher’s exact test, p = 0.057). Moreover, samples that failed the quality test were significantly more likely to fail the observational test of this study (p < 0.05, Table 2). This outcome indicates that appropriate storage conditions and packaging may help prevent deterioration in the quality and bio-availability of medicines. Some previous studies also reported damaged packaging as a prominent characteristic of counterfeit and substandard medicines [15,29].

Clavulanic acid is volatile and unstable when exposed to high temperatures and high pH [30]. In addition, clavulanic acid is hygroscopic; therefore, 30% RH or less is desirable for storage [31,32]. The harmful effects of experimentally introduced packaging defects on amoxicillin–clavulanic acid revealed here were in accord with recent studies: clavulanic acid was the main factor in the degradation of mixtures [33-35]. Our results show that several samples failed the quantity and content uniformity tests because of low clavulanic acid contents. Degradation of clavulanic acid may occur during manufacturing, in distribution, or in storage at the pharmacy. Packaging practices and materials have been reported to contribute to the degradation of medicines in several studies [19,36]. Improvement and standardization of packaging, as well as strict compliance with good distribution and pharmacy practices, may help to maintain the quality of medicines for the end user.

Most of the samples that failed the quality tests in this study were generic. The circulation of substandard generic amoxicillin-clavulanic acid was reported in two recent studies in other countries [37,38]. Some reports suggest that different coatings and additives with similar active ingredients are used to make medicines bio-available [39]. Therefore, the low dissolution rate of amoxicillin observed in this study might be due to the additives used in generic formulations. There is evidence that poor-quality medicines commonly fail dissolution tests, which is in accord with our findings [40].

### Limitation

We did not measure potassium content; this omission could be considered a limitation of this study. This decision was made because of the low number of tablets in each samples and limited resource. Analysis of potassium might provide important clues as to whether the degradation of clavulanic acid occurred in the supply chain or at the manufacturer. One objective of this study is to check quality authenticity and pharmacopoeial quality within the framework of limited collected samples and resource and quickly report these values for necessary measures, even in cases of incomplete investigation of the cause and source of such medicines. Another limitation of this study was the absence of liquid formulations such as syrups and suspensions due to our selection criteria. Further study is required to evaluate these commonly used products.

## Conclusion

This study shows that the condition of packaging is an important factor in maintaining the quality of amoxicillin–clavulanic acid products. To ensure quality, manufacturers must comply with GMP requirements. Distributors and service providers also need to be very stringent in adhering to quality assurance and standard operating procedures. It may not be possible to completely eliminate antibiotic resistance; however, through the improved management and rational use of antibiotics, we may be able to delay the process.

## Competing interests

The authors declare that they have no competing interests.

## Authors’ contributions

MHK, KH, TS, NN, TT and KK participated in design, fieldwork and documentation; MHK, KH, TS, NN, LCCC, NY, HT and KK participated in data analysis; MHK, KH, TS, NN, NY, HT, TT and KK participated in the interpretation of results. MHK wrote the first draft. All authors participated in a critical review of the manuscript and approved the submitted version. Additionally, all authors have read and approved the final manuscript.

## Pre-publication history

The pre-publication history for this paper can be accessed here:

http://www.biomedcentral.com/2050-6511/14/33/prepub
